# Effects of Innovation, Total Quality Management, and Internationalization on Organizational Performance of Higher Education Institutions

**DOI:** 10.3389/fpsyg.2022.869638

**Published:** 2022-04-14

**Authors:** Joaquín Texeira-Quiros, Maria do Rosário Justino, Marina Godinho Antunes, Pedro Ribeiro Mucharreira, António de Trindade Nunes

**Affiliations:** ^1^Department of Economic and Business Science, Autonomous University of Lisbon, Lisbon, Portugal; ^2^Lisbon Accounting and Business School, Lisbon Polytechnic, Lisbon, Portugal; ^3^Institute of Education, University of Lisbon, Lisbon, Portugal; ^4^Department of Financial Economics and Accounting, University of Extremadura, Badajoz, Spain

**Keywords:** quality, innovation, internationalization, organizational performance, higher education institutions (HEIs)

## Abstract

The aim of this research is to analyze the effects of innovation strategies, Total Quality Management (TQM) dimensions, and internationalization strategies that Higher Education Institutions (HEIs) might adopt, and their effects on their organizational performance. Due to globalization and the constant changes and demands that have taken place today, HEIs are forced to seek new quality assurance instruments in higher education, to ensure greater competitiveness in the markets and their survival. To examine the association between the independent variables, namely, TQM dimensions, innovation strategies, and internationalization strategies with the dependent variable, that is organizational performance of HEIs, we have chosen to use multiple linear regression analysis. A nine-predictor multiple linear regression model was proposed. The nine predictor variables are Communication, Involvement/teacher empowerment, Development/Teacher training, Continuous improvement, Leadership/Administration’s Commitment, Data analysis/Measurement of results, Focus on students, Innovation Strategies, and Internationalization strategy. We conclude that some TQM variables have a significant association with the organizational performance of HEIs, namely, Involvement/teacher empowerment, and Development/teacher training. On the other hand, also the Innovation strategies and Internationalization strategy have a significant association with the organizational performance of HEIs. This research is of enormous importance for the study of HEIs, considering their role in the development of any country and its impact on society as creators of knowledge and science. Since these institutions increasingly must deal with extremely competitive market environments, knowledge of the factors that can assist in increasing the organizational performance of HEIs is of great relevance.

## Introduction

Education and science have proved to be factors with a profoundly significant impact on the economy and society in general, and their level of development in a given country significantly affects people’s quality of life and the development of that country itself, as well as an international perspective.

Science and qualified people are recognized around the world as the decisive factor in achieving the goals of internationalization of any company, and consequently, of the educational process, which leads to the search for a more competitive, dynamic, and capable education to ensure sustainable growth. In this context, higher education represents a critical factor in the development of this capital and innovation, playing a fundamental role in the success and sustainability of economic development.

Higher education institutions (HEIs) are forced to look for new quality instruments in higher education, as they are conditioned by globalization and the constant changes that have taken place around the world. Nowadays, HEIs are more diversified and closer to a model characterized by supply and demand. Achieving quality is the most important element of efficient and effective higher education. The quality of teaching and learning has proved to be a strategic issue of enormous importance in higher education systems (Seyfried and Pohlenz, 2018).

Nowadays, HEIs face several challenges, such as rapid and urgent technological changes in education, internationalization, and competition strategies, as well as a constant concern to control costs and financing (Laurett and Mendes, 2019). Thus, these institutions must meet and exceed the expectations of their stakeholders (Dumond and Johnson, 2013), leading them to adopt different strategies, such as Total Quality Management (TQM) and innovation (Chen et al., 2009). HEIs can and should improve their processes and services through the effective implementation of quality (Iqbal et al., 2018), as they play a key role in the economic growth and development of societies since they are the ones who develop, promote, and disseminate new ideas and knowledge. On the other hand, and according to Al-Husseini and Elbeltagi (2016), innovation is also fundamental in the definition of HEI strategies, as it can help in program revisions and improve the institutions’ problem-solving capacity.

Different approaches have been adopted with regard to quality management in universities, such as self-assessment and external assessment of institutions, accreditation systems, or TQM models (Goldberg and Cole, 2002). In general, the implementation of a relevant TQM framework that meets the institutions’ mission and objectives should be the first step to improve the quality of products and services provided by HEIs (Venkatraman, 2007). TQM represents a strategic option and an integrated management philosophy for organizations, which allows them to achieve their goals effectively and efficiently, and achieve sustainable competitive advantage. According to Todorut (2013), TQM can define HEI strategies with regard to desirable services and customer satisfaction. Academic programs are the main product of HEIs to attract and satisfy all stakeholders, especially students (Bayraktar et al., 2008).

From another perspective, it is also relevant in this investigation to introduce the concept of innovation. Higher education systems must seek to innovate (Barber et al., 2013), that is, HEIs must rethink their work model and create knowledge that can be marketed in new products and services. Educational innovations include pedagogical innovation, scientific and methodological innovation, educational, and technological innovation. HEIs that have opted for innovation-based development become competitive leaders in the educational market.

Investigations developed with a focus on innovation and the performance of HEIs have shown that innovation plays a key role for this type of institution, as it allows for continuous improvement in their performance (Jaskyte, 2004; Chen and Chen, 2012). According to Jaskyte (2004), HEIs need to invest in product and process innovation to improve their educational performance, thus achieving competitive advantages over other institutions (Chen and Chen, 2012).

Regarding the internationalization of HEIS, several researchers have focused on quality issues in HEIS, as it is an increasingly competitive context, both nationally and internationally (Moogan et al., 2001; Nasim et al., 2020). HEIs have increasingly invested in their internationalization strategies, in order to obtain greater recognition and notoriety across borders, as a destination for higher studies and research. The internationalization of education is the highest stage of international relations between HEIs and is today considered a means of improving and promoting the quality of teaching, learning, and research. The acquisition of knowledge, the mobilization of talent, applied research, and curricula shared by partner institutions are considered the main benefits of the internationalization of higher education (Telford and Masson, 2005; Yeo, 2008).

## Theoretical Framework

### Total Quality Management in Higher Education

Total quality management is a management philosophy, transversal to the entire organization, and which incorporates all stakeholders in its model structure, seeking the continuous improvement of organizational performance and customer satisfaction. Several studies that have focused on the role of TQM in competitive advantage have shown that its implementation promotes better performance and greater competitiveness (Terziovski and Samson, 1999; Zhang, 2000; Texeira-Quirós and Justino, 2013; Texeira-Quirós et al., 2013; Antunes et al., 2017, 2018a). The application of TQM principles in HEIS has recently emerged, framed in new realities that began to recognize HEIs as profitable organizations (Antunes et al., 2018a,^2020^, ^2021^). In this framework, there is a need for differentiation from other HEIs, justifying the increasing implementation of TQM practices (Nasim et al., 2020).

Due to globalization and internationalization, there are often constant changes that arise and occur very quickly at different levels. This means that, in the business environment and taking into account the organizational dynamics, they must react must react strategically to these changes, if they want to remain competitive in their activity. In this perspective, several studies have found a positive relationship between quality management practices and performance, such as the studies of Calvo-mora et al. (2005), Sayeda et al. (2010), and Psomas and Antony (2017).

Investment in education is crucial to promoting economic growth and development. Education is present in all aspects of society, being one of its constitutive elements. This means that education is related to the political, economic, scientific, and cultural context of a given society, that is, education reinforces the critical capacity of the individual and makes it possible to assess the degree of development of a society. In this way, it is quite evident that the quality of education provided in the HEIs is assumed as a fundamental element of the management of the HEIs (Reed et al., 1996; Rahman, 2004). Quality plays an important role in customer satisfaction and in the recognition of institutions in the market. However, this task assumes some complexity when it comes to evaluating educational systems and HEI processes, as they are intangible assets (Cabrito, 2009).

Total quality management is based on a principle of continuous improvement. Although the TQM principles were initially used in the industrial sector, the implementation of TQM practices is also applicable to HEIs, having the potential to improve quality in educational institutions (Hendricks and Singhal, 2001; Martinez-Costa and Jiménez-Jiménez, 2008). The implementation of TQM practices at HEIs has been driven by increased competition, the adaptation to the educational environment in constant evolution, and by meeting the expectations of all stakeholders (Bayraktar et al., 2008).

The current context of higher education emphasizes the importance of the TQM principles in the education system. However, the existing literature has highlighted the fact that educational institutions have lagged other organizations, regarding the culture of total quality (Antunes et al., 2018b). These authors defined TQM’s philosophy as the culture of an organization committed to customer satisfaction through continuous improvement, and this culture may vary from one country to another and between different industries.

The existing literature on the implementation of TQM in HEIs highlights different aspects, such as customer focus and orientation, leadership, teamwork, process-oriented approach, including design and process improvement, and the use of TQM tools and techniques. According to Flynn et al. (1994), a critical step in the implementation of TQM is the identification and orientation for the customer. This raises the question of who the customer is when referring to HEIs. According to Houston (2007), in the case of HEIs, students should be considered customers.

The conclusions about the usefulness of implementing TQM in HEIs are divergent (Meirovich and Romar, 2006). Some authors believe that the principles of TQM are equally applicable in HEIs (Sirvanci, 2004), and that they are compatible with this type of organizations (Helms and Key, 1994; Venkatraman, 2007), while others argue that the principles of TQM are only marginally useful when it comes to a dynamic and constantly changing environment, as is the case with current higher education (Houston, 2007).

Many of the researches that have focused on TQM’s repercussions on competitive advantage have shown that their presence leads to improved performance and increased competitiveness (Terziovski and Samson, 1999; Zhang, 2000; Texeira-Quirós and Justino, 2013; Texeira-Quirós et al., 2013; Antunes et al., 2017, 2018b,^2021^). In this sense, the following hypotheses of the research were defined:

H1: *Communication has a significant association with the organizational performance of HEIs.*
H2: *Involvement/teacher empowerment has a significant association with the organizational performance of HEIs.*
H3: *Development/teacher training has a significant association with the organizational performance of HEIs.*
H4: *Continuous improvement has a significant association with the organizational performance of HEIs.*
H5: *Leadership/administration’s commitment has a significant association with the organizational performance of HEIs.*
H6: *Data analysis/measurement of results has a significant association with the organizational performance of HEIs.*
H7: *Focus on students has a significant association with the organizational performance of HEIs.*

### Innovation in Higher Education

The higher education sector is pressured by a dynamic environment that is characterized by rapid technological changes and increased demand (Mathew, 2010) to which it is necessary to give a strategic response (Fullwood et al., 2013). Essentially, innovation provides for the exploitation of existing business opportunities as well as new opportunities that may arise in the future. And, once again mentioned, as markets, technologies, and trends are constantly changing, innovation strategies are considered the main factor that helps companies stay ahead of their rivals. Obendhain and Johnson (2004) refer that HEIs are important, as they are producers of innovation through the creation of products and services. Organizations increasingly focus on innovation as a key factor for success and competitive advantage.

According to Sciarelli et al. (2020), quality practices improve innovation and organizational performance, while innovation positively impacts organizational performance, following the studies of De Jong and Hartog (2007), which described innovation as the creation of new ideas, products, and processes, having concomitant effects on performance. According to these authors, innovation plays a key role in improving business performance and, therefore, HEIs must constantly innovate in all aspects of their business operations so that they can compete and survive in the competitive market. Other authors, like Damanpour (2009), stated that innovation is important because it helps organizations to adapt and respond to environmental and technological changes and Nusair et al. (2012) refer that innovation consists in the development and implementation of new ideas, methods, and procedures to achieve the organization’s objectives effectively. Innovation means the perceived novelty of the idea from the point of view of individuals. Innovation can be anything related to the introduction of a new product, process, technology, business system, business model, service, or marketing activity (Han et al., 2013). An innovation strategy can have positive effects on HEIs performance, however, a company will not be able to benefit from the advantages of innovation if there is no defined organizational structure to follow this strategy. As HEIs continue to make progress on various criteria, they need to develop innovative solutions in response to changing expectations about their performance, which suggests HEIs should foster innovation. Innovation may reflect a significant impact on the institution’s performance by enabling a better position in the market, which, in turn, can give it a competitive advantage and superior performance.

Innovation has been considered as a strategic driver to take advantage of new opportunities (Teece, 2000; Gatignon et al., 2002; Lloréns-Montes et al., 2005; Hurmelinna-Laukkanen et al., 2008). Therefore, innovation has sparked the interest of many researchers to identify their push factors (Becheikh et al., 2006). Dannels (2002) pointed out that innovation translates competencies into new products and processes, allowing organizations to obtain a competitive advantage. Product and process innovation can increase an organization’s adaptability to environmental changes and tends to be present in organizations where problem-solving and creativity take place (Tsai et al., 2001).

Innovation in HEIs is considered the main driver of economic and social development, making the study of these two types of innovation in higher education environment essential. Research on product innovation shows that it is of great interest because it is a critical point prior to product success, which in turn is associated with organizational success (Valencia et al., 2010). The quality and opportunity that characterize competition based on innovation are extremely important for the survival of HEIs. Institutions that opt for innovation-based development become competitive leaders in the educational market, and the ones that more easily adapt to changes and respond positively to the various challenges. Considering this, the following research hypothesis is considered:

H8: *Innovation strategies have a significant association with the organizational performance of HEIs.*

### The Role of Internationalization in Higher Education

The internationalization of HEIs can be defined as the process of integration of the institution and its main stakeholders, such as students, teachers, and employees, in a world in rapid transformation and globalization. Internationalization contributes to the formation of dynamic citizens, capable of working in foreign and culturally diverse environments (Altbach and Knight, 2007; Denson and Zhang, 2010). In this way, internationalization strategies derive from the growing need to contribute to the evolution of global labor markets and, consequently, to foster international learning.

Internationalization leads HEIs to follow international standards, thus offering national and international students greater skills and opportunities, and also being more attractive (Luijten-Lub, 2007). Also according to Taylor (2004), internationalization is perceived as a beneficial effect on the quality of educational services, by stimulating compliance with international levels of quality, facilitating the development of joint programs, through the involvement of foreign teachers, and the organization extracurricular activities (Van der Wende, 2007).

Concerning internationalization, it is worth mentioning the growing international partnerships in terms of research projects and scientific publications, and the positive externalities that result from this (Cerdeira et al., 2021; Civera et al., 2021). On the other hand, internationalization is essential to complement resources, skills, and knowledge to discover innovative solutions and respond to today’s global challenges (Knight, 2004; Fielden, 2006). Internationalization can be a very important strategy to increase the prestige of HEIs and their recognition at the national and international levels (De Wit, 2002; Knight, 2004; Chan and Dimmock, 2008). For example, most Portuguese HEIs, public or private, have an International Relations Office. This unit operates under the responsibility of each HEI, and coordinates, monitors, and supports the development of all initiatives related to internationalization, namely in the sphere of academic cooperation and student and professor mobility.

It is no coincidence that the majority of scientifically renowned HEIs are often also highly internationalized (Horn et al., 2007), which means that highly internationalized HEIs tend to be seen as high quality in the same way. According to Knight (2004), the development of international strategic alliances aims to create synergies, capitalize efforts and collective investments, and internationalization can likewise foster networks of researchers across national borders, resulting in positive effects for institutions. Thus, the following research hypothesis is considered:

H9: *Internationalization strategy has a significant association with the organizational performance of HEIs.*

**FIGURE 1:**
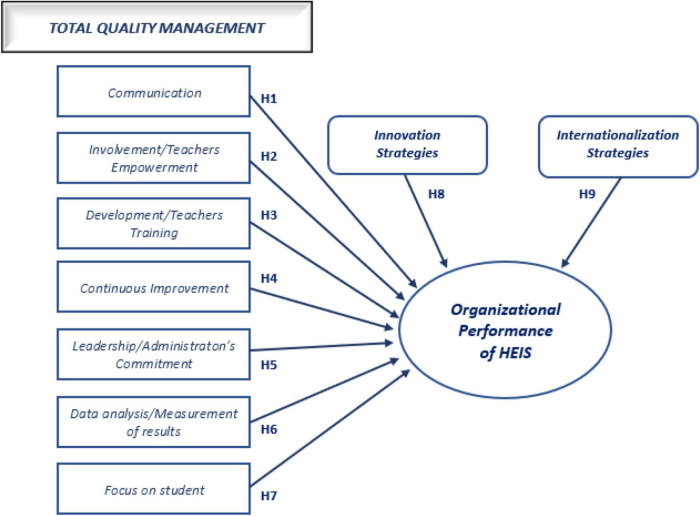
Conceptual model and research hypotheses.

Considering the theoretical framework presented, the conceptual model and research hypotheses are shown in Figure 1.

## Empirical Research

### Data Collection and Sample Characterization

Data collection was done through a questionnaire, elaborated with closed questions, using a five-point Likert scale to evaluate the respondents’ conceptions about the dimensions considered, where 1 represents “strongly disagree” and 5 represents “strongly agree.” For the characterization of respondents and institutions, nominal and ordinal scales were used.

The questionnaire was sent *via* email, which contained a brief explanation of the investigation and an access link to it. The questionnaire was addressed to higher education teachers, namely from public and private universities and polytechnic institutes who, in addition to their teaching activity, simultaneously perform other institutional functions, such as belonging to the presidency or rectory, being members of the scientific council, members of the pedagogical council, directors of undergraduate or master’s degrees, etc. Data collection was carried out between October and December of 2019, and 316 valid questionnaires were received.

Concerning the characteristics of the respondents, 170 were female (53.8%) and 146 male (46.2%), only one respondent was under 30 years old (0.3%), 32 were between 31 and 30 years old (10.1%), 122 were between 41 and 50 years old (38.6%), 113 were between 51 and 60 years old (35.8%), and 48 were over 61 years old (15.2%). Regarding the years of academic experience in higher education, 6 respondents had less than 5 years (1.9%), 32 had between 5 and 10 years of activity (10.1%), 89 had between 11 and 20 years (28.2%), and 189 were over 20 years of academic experience (59.8%).

### Data Analysis

To examine the association between TQM dimensions, innovation strategies, and internationalization strategies with the organizational performance of HEIs, a multiple linear regression analysis was used. According to Hair et al. (1998), it is a practical statistical tool that examines the linkages between a set of independent variables with one dependent variable. Thus, a nine-predictor multiple linear regression model was proposed. The nine predictor variables are Communication (X1), Involvement/teacher empowerment (X2), Development/Teacher training (X3), Continuous improvement (X4), Leadership/Administration’s Commitment (X5), Data analysis/Measurement of results (X6), Focus on students (X7), Innovation Strategies (X8), and Internationalization strategy (X9). The equation of the proposed multiple linear regression model is illustrated as follows:


Y⁢(P⁢1)=b⁢0+b⁢1⁢(X⁢1)+b⁢2⁢(X⁢2)+b⁢3⁢(X⁢3)+b⁢4⁢(X⁢4)+b⁢5⁢(X⁢5)+b⁢6⁢(X⁢6)+b⁢7⁢(X⁢7)+b⁢8⁢(X⁢8)+b⁢9⁢(X⁢9)+ε

where:

*Y (P1)* = *Dependent variable (organizational performance of HEIs), b0* = *Constant*,ε = *Error.*

First, the reliability test was performed for the variables involved in the investigation using Cronbach’s Alpha. All values were greater than 0.67, which means that there is a good to excellent internal consistency (Table 1). After obtaining these values, namely the values of internal consistency, the scores of each of the dimensions were calculated adding the questions belonging to each one of them. That is, Cronbach’s Alpha values are high, so it makes sense to calculate the scores for each dimension using all the questions that were chosen to define this dimension. For the calculation of scores, they were considered, in the case of “Internationalization strategies” (Q9 + Q10 + Q11 + Q12 + Q13)/5, and so on for the others.

**TABLE 1:** Cronbach’s Alpha.

Dimensions	Question number	Cronbach’s Alpha
Internationalization strategies	Q9, Q10, Q11, Q12, Q13	0.843
Organizational performance		0.858
* Operational performance	Q19, Q20, Q21, Q22, Q23, Q24	0.672
* Market performance	Q25, Q26, Q27, Q28, Q29, Q30	0.785
* Financial performance	Q31, Q32, Q33, Q34, Q35, Q36	0.758
**TQM dimensions**		
* Design/conception of programs	Q47, Q48, Q49, Q50	0.804
* Involvement/teacher empowerment	Q51, Q52, Q53, Q54	0.884
* Development/teacher training	Q55, Q56, Q57, Q58	0.910
* Continuous improvement	Q59, Q60, Q61, Q62	0.915
* Leadership/administration’s commitment	Q63, Q64, Q65, Q66	0.931
* Data analysis/measurement of results	Q67, Q68, Q69, Q70	0.899
* Focus on students	Q71, Q72, Q73, Q74	0.785
Innovation strategies		0.937
* Products and services–courses	Q75, Q76, Q77, Q78, Q79	0.929
* Processes	Q80, Q81, Q82, Q83, Q84	0.924

Regarding the characterization of dependent and independent variables, Table 2 shows the descriptive statistics of the variables under study, with the variable “Focus on Students” having the highest average of *M* = 3.86 and the variables “Involvement/teacher empowerment” and “Development/teacher training” those with the lowest average value (*M* = 2.98 and *M* = 2.97, respectively).

**TABLE 2:** Descriptive statistics of the variables under study.

	N	Min	Max	Mean	Std. Deviation
Organizational performance (Y)	316	1.78	5.00	3.43	0.54
Design/conception of programs (X1)	316	1.00	5.00	3.28	0.85
Involvement/teacher empowerment (X2)	316	1.00	5.00	2.98	0.97
Development/teacher training (X3)	316	1.00	5.00	2.97	1.02
Continuous improvement (X4)	316	1.00	5.00	3.23	0.90
Leadership/administration’s commitment (X5)	316	1.00	5.00	3.31	1.04
Data analysis/measurement of results (X6)	316	1.00	5.00	3.22	0.92
Focus on students (X7)	316	1.00	5.00	3.86	0.78
Innovation strategies (X8)	316	1.00	5.00	3.33	0.84
Internationalization strategies of HEIs (X9)	316	1.40	5.00	3.71	0.77

Pearson’s correlation between the variables was also performed, verifying that all independent variables have a significant and positive correlation with the dependent variable (Table 3).

**TABLE 3:** Pearson’s correlation.

	Y	X1	X2	X3	X4	X5	X6	X7	X8	X9
Y	1	0.614**	0.675**	0.674**	0.627**	0.658**	0.633**	0.537**	0.674**	0.585**
X1		1	0.693**	0.639**	0.728**	0.702**	0.641**	0.645**	0.724**	0.499**
X2			1	0.764**	0.755**	0.758**	0.751**	0.623**	0.707**	0.537**
X3				1	0.713**	0.744**	0.717**	0.608**	0.677**	0.553**
X4					1	0.774**	0.744**	0.600**	0.719**	0.491**
X5						1	0.767**	0.634**	0.752**	0.529**
X6							1	0.629**	0.703**	0.538**
X7								1	0.664**	0.416**
X8									1	0.477**
X9										1

**Correlation is significant at the 0.01 level (2-tailed).

Then, the multiple linear regression model was estimated. The variable selection method chosen was Stepwise, where the first independent variable to enter is the one with the highest correlation coefficient (0.675), which corresponds to the variable “Involvement/teacher empowerment.”

Table 4 presents a summary of the model showing the multiple correlation coefficient *R*, the determination coefficient *R*^2^, and the adjusted determination coefficient. Approximately 60% of the total variability of the dependent variable “Organizational Performance” (*R*^2^adj = 0.592) is explained by the independent variables presented in the model.

**TABLE 4:** Model summary.

Model	*R*	*R* square	Adjusted *R* square	Std. error of the estimate	Change statistics					Durbin-Watson
					*R* square change	*F* change	df1	df2	Sig. *F* change	
1	0.675^a^	0.455	0.454	0.40133	0.455	262.673	1	314	0.000	
2	0.730^b^	0.533	0.530	0.37230	0.077	51.863	1	313	0.000	
3	0.763^c^	0.582	0.578	0.35290	0.049	36.373	1	312	0.000	
4	0.773^d^	0.597	0.592	0.34698	0.015	110.729	1	311	0.001	10.706

*^a^Predictors: (Constant), Involv_X2.*

*^b^Predictors: (Constant), Involv_X2, Innovation_X8.*

*^c^Predictors: (Constant), Involv_X2, Innovation_X8, Intern_X9.*

*^d^Predictors: (Constant), Involv_X2, Innovation_X8, Intern_X9, Develop_X3.*

The Anova test analyzes the significance of the model and given that *p* < 0.05 [*F*(4,311) = 115.115; *p* < 0.001] was obtained, it is concluded that the model is adequate (Table 5).

**TABLE 5:** ANOVA test.

Model		Sum of squares	df	Mean square	*F*	Sig.
1	Regression	42,307	1	42,307	262.673	0.000^a^
	Residual	50,574	314	0.161		
	Total	92,881	315			
2	Regression	49,496	2	24,748	178.543	0.000^b^
	Residual	43,385	313	0.139		
	Total	92,881	315			
3	Regression	54,025	3	18,008	144.605	0.000^c^
	Residual	38,855	312	0.125		
	Total	92,881	315			
4	Regression	55,438	4	13,859	115.115	0.000^d^
	Residual	37,443	311	0.120		
	Total	92,881	315			

*^a^Predictors: (Constant), Involv_X2.*

*^b^Predictors: (Constant), Involv_X2, Innovation_X8.*

*^c^Predictors: (Constant), Involv_X2, Innovation_X8, Intern_X9.*

*^d^Predictors: (Constant), Involv_X2, Innovation_X8, Intern_X9, Develop_X3.*

Table 6 shows the results of multiple linear regression. Thus, of the nine variables that initially entered the model, only four were significant and all had a positive coefficient, namely, “Involvement/teacher empowerment” (X2) (B1 = 0.105; *t* = 3.031; *p* = 0.003), “Innovation Strategies” (X8) (B2 = 0.189; *t* = 5.455; *p* < 0.001), “Internationalization Strategy” (X9) (B3 = 0.163; *t* = 5.198; *p* < 0.001), and “Development/Teacher training” (X3) (B4 = 0.110; *t* = 3.425; *p* = 0.001).

**TABLE 6:** Multiple linear regression results.

Model		Unstandardized coefficients		Standardized coefficients	t	Sig.	95.0% confidence interval for B		Collinearity statistics	
							
		B	Std. error	Beta			Lower bound	Upper bound	Tolerance	VIF
1	(Constant)	2.304	0.073		31.451	0.000	2.160	2.449		
	Involv_X2	0.379	0.023	0.675	16.207	0.000	0.333	0.425	1.000	1.000
2	(Constant)	1.922	0.086		22.269	0.000	1.752	2.091		
	Involv_X2	0.223	0.031	0.397	7.255	0.000	0.162	0.283	0.500	2.001
	Innovation_X8	0.255	0.035	0.394	7.202	0.000	0.185	0.324	0.500	2.001
3	(Constant)	1.516	0.106		14.305	0.000	1.307	1.724		
	Involv_X2	0.163	0.031	0.290	5.309	0.000	0.103	0.224	0.448	2.232
	Innovation_X8	0.221	0.034	0.342	6.512	0.000	0.154	0.288	0.486	2.056
	Intern_X9	0.187	0.031	0.265	6.031	0.000	0.126	0.248	0.692	1.444
4	(Constant)	1.559	0.105		14.858	0.000	1.353	1.766		
	Involv_X2	0.105	0.035	0.187	3.031	0.003	0.037	0.173	0.341	2.936
	Innovation_X8	0.189	0.035	0.292	5.455	0.000	0.121	0.258	0.451	2.217
	Intern_X9	0.163	0.031	0.231	5.198	0.000	0.101	0.224	0.657	1.523
	Develop_X3	0.110	0.032	0.206	3.425	0.001	0.047	0.173	0.360	2.779

Thus, the adjusted model is:


$$Organizational\;performance\;of\;HEI^{\prime }s=1,559+0,105*$$

Involvement/teacherempowerment(X2)+0,189*

I⁢n⁢n⁢o⁢v⁢a⁢t⁢i⁢o⁢n⁢S⁢t⁢r⁢a⁢t⁢e⁢g⁢i⁢e⁢s⁢(X⁢8)+0,163*I⁢n⁢t⁢e⁢r⁢n⁢a⁢t⁢i⁢o⁢n⁢a⁢l⁢i⁢z⁢a⁢t⁢i⁢o⁢n

s⁢t⁢r⁢a⁢t⁢e⁢g⁢y⁢(X⁢9)+0,110⁢D⁢e⁢v⁢e⁢l⁢o⁢p⁢m⁢e⁢n⁢t/T⁢e⁢a⁢c⁢h⁢e⁢r⁢t⁢r⁢a⁢i⁢n⁢i⁢n⁢g⁢(X⁢3)+ε.

Concerning the interpretation of the model, its values mean that the increase of one unit in the variable “Involvement/teacher empowerment” results in an average increase of 0.105 units in the value of the dependent variable “Organizational performance,” the increase of one unit in the variable “Development/teacher training” implies the increase of 0.110 units in the value of “Organizational performance,” an increase of one unit in “Innovation strategies” implies an increase of 0.189 units in the value of “Organizational performance,” and an increase of one unit in the variable “Internationalization strategy” implies an increase of 0.163 units in the value of “Organizational performance.”

Thus, analyzing the hypotheses of the study, the following were verified *H2: Involvement/teacher empowerment has a significant association with organizational performance of HEIs; H3: Development/teacher training has a significant association with organizational performance of HEIs; H8: Innovation strategies have a significant association with organizational performance of HEIs;* and *H9: Internationalization strategy has a significant association with organizational performance of HEIs.*

After the results of the multiple regression model, it was necessary to verify the assumptions of the model studied. These assumptions consist in the analysis of the residues, that is, to verify if they follow a normal distribution, if they have a zero average, if they are independent, homoscedastic (constant variance), and verify the existence of multicollinearity.

Regarding the verification of the normal distribution of residues, Table 7 shows the Kolmogorov–Smirnov and Shapiro–Wilk normality tests for standardized residues. As the values obtained are *p* = 0.200 and *p* = 0.963, respectively, it is concluded that the residues follow a normal distribution.

**TABLE 7:** Tests of normality of Kolmogorov–Smirnov and Shapiro–Wilk.

	Kolmogorov–Smirnov^a^			Shapiro–Wilk		
		
	Statistic	df	Sig.	Statistic	df	Sig.
Standardized residual	0.024	316	0.200*	0.998	316	0.963

*This is a lower bound of the true significance.

*^a^Lilliefors significance correction.*

The Durbin–Watson statistic assesses the covariance of the residues, that is, their autocorrelation. Values close to 2 indicate no residual autocorrelation, values close to zero indicate a positive correlation, and values close to 4 indicate a negative autocorrelation. Given that the Durbin–Watson statistic obtained was 1.706 (Table 4), a value that is close to 2, it allows us to conclude that there is no autocorrelation of the residues.

**FIGURE 2:**
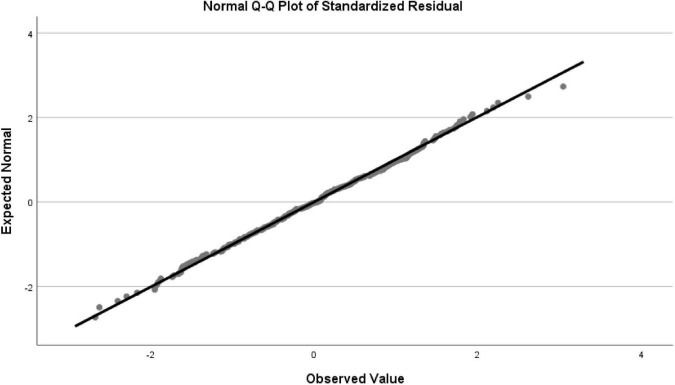
QQ plot graph of the normality of the residues.

**FIGURE 3:**
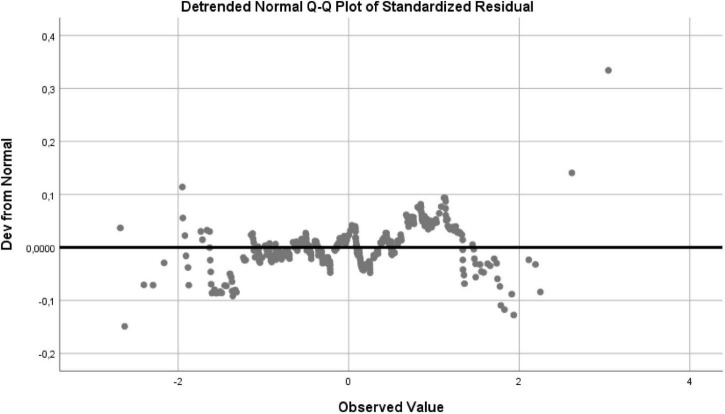
Detrended QQ plot graph of the normality of the residues.

Figure 2 refers to the QQ Plot Graph of the normality of the residues, which allows us to verify that the closer and on top of the line it means that the distribution of the residues is normal. In Figure 3 we have the Detrended QQ Plot graph of the normality of the residuals, where we can see that most of the observations are distributed around the horizontal line, meaning that the distribution of the residuals is normal.

**FIGURE 4:**
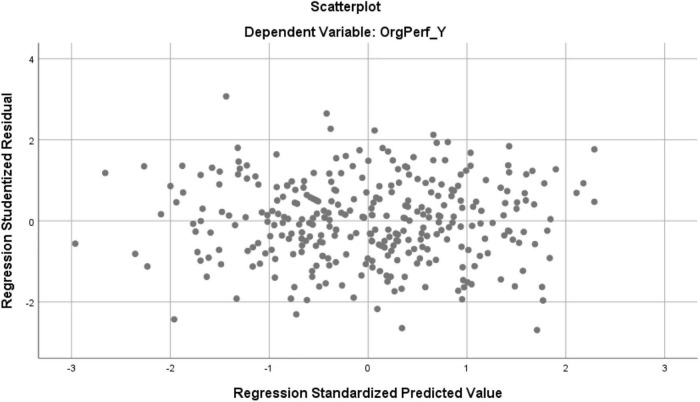
Graph of the analysis of the variance of the residues.

To verify homoscedasticity, that is, to analyze whether errors have constant variance, a graph of Studentized residuals and Standardized Predicted values was performed. Looking at the graph (Figure 4). It appears that the points are randomly distributed around 0, with no behavior or trend, concluding that the variance of the residuals is constant.

Finally, in order to assess whether there is multicollinearity, that is, whether the independent variables are correlated, the Tolerance value ranging from 0 to 1 was calculated. If this value is close to zero, we will have multicollinearity problems. The inverse of Tolerance is called VIF (Variance Inflation Factor). The value usually considered as the limit above which there is multicollinearity is 10. Table 6 shows, in the VIF and Tolerance columns, that all VIF values are less than 10 and no tolerance value is close to 0. Thus, it is concluded that there is no Multicollinearity.

## Conclusion

This research is of enormous importance for the study of HEIs, considering their role in the development of any country and its impact on society as creators of knowledge and science. Since these institutions increasingly must deal with extremely competitive market environments, knowledge of the factors that can assist in increasing the organizational performance of HEIs is of great relevance. TQM has come to be recognized as a tool that allows to obtain a significant competitive edge in the global market (Helms and Key, 1994; Sirvanci, 2004; Venkatraman, 2007). Concerning our results about the TQM dimensions, we can conclude that Involvement/teacher empowerment and Development/teacher training have a significant association with the organizational performance of HEIs. Although there is not a statistically significant relationship with all dimensions of TQM, only with two, these results are in line with previous studies, such as (Terziovski and Samson, 1999; Texeira-Quirós and Justino, 2013; Antunes et al., 2017, 2018b), which had evidenced a positive relationship between TQM and the organizational performance of organizations.

On the other hand, innovation strategies can provide organizations with certain benefits, which often justify the investments made to develop new products and services in order to reach new markets or the development of technologies, processes, and procedures to support the production of these products and the provision of services. Concerning innovation strategies, our research showed a significant association with organizational performance of HEIs, results that are supported by the studies of Dannels (2002), De Jong and Hartog (2007), and Nusair et al. (2012), who referred that innovation strategies allow institutions to obtain a competitive advantage, thus translating into better organizational performance.

Finally, the issue of internationalization has, in recent years, been playing a leading role, not only in Europe but throughout the world. With regard to the internationalization of HEIs, several authors have analyzed the question of their effects on these institutions in order to obtain a sustainable competitive advantage (Ebrahimi and Sadeghi, 2014), and as a way to respond to today’s global challenges (Knight, 2004; Fielden, 2006; Chan and Dimmock, 2008). Our results allow to conclude that internationalization strategies have a significant association with the organizational performance of HEIs.

## Data Availability Statement

The original contributions presented in the study are included in the article/supplementary material, further inquiries can be directed to the corresponding author/s.

## Ethics Statement

Ethical review and approval was not required for the study on human participants in accordance with the local legislation and institutional requirements. Written informed consent from the patients/participants was not required to participate in this study in accordance with the national legislation and the institutional requirements.

## Author Contributions

All authors listed have made a substantial, direct, and intellectual contribution to the work, and approved it for publication.

## Conflict of Interest

The authors declare that the research was conducted in the absence of any commercial or financial relationships that could be construed as a potential conflict of interest.

## Publisher’s Note

All claims expressed in this article are solely those of the authors and do not necessarily represent those of their affiliated organizations, or those of the publisher, the editors and the reviewers. Any product that may be evaluated in this article, or claim that may be made by its manufacturer, is not guaranteed or endorsed by the publisher.
